# Suits for Malpractice, so Common as to Constitute an Epidemic

**Published:** 1878-12

**Authors:** 


### Suits for Malpractice,

GROWING SO COMMON AS TO CONSTITUTE AN EPIDEMIC IN THE STATE OF NEW YORK.

We desire to call attention to some of the causes, symptons and means of cure of this alarming disease now so prevalent. The remote causes are, briefly, that the verdict of a petit jury is no index of right, honesty, or law, is indeed a bigoted, idiotic or imbecile prejudiced expression of sympathy and nothing more. The decision of a Court in New York is no more of a guide to truth and right, than the falling of a stick was to a lost traveler in our ancient forests. Such a chance is better, safer, more creditable, more reasonable and in all respects a more civilized mode of determining or deciding the merits or demerits of our professional conduct than our courts of law. We are pained to believe that an institution so hoary with age, and so respectably related to society, is a barbarism as atrocious in civil cases as hanging a witch or burning a lunatic, practiced hundreds of years ago. We boast of our civilization, but would it not be as becoming to go out and put on "sackcloth and ashes," and weep that we were born so soon—so long before the dawn of civilization.

The medical profession of this country has influence enough if rightly applied, to correct in some degree the barbarism of our present code,—to insure in medical cases a jury of physicians or of selected scientific men—a jury from our peers, in the phraseology of law. But as reformation must meet opposition and requires time, we are happy to announce that Physicians of Chautauqua Co., we understand, have organized for self-defense, in suits for mal-practice. We quote from a private letter from one of the most experienced and influential physicians and surgeons of the county.

"We have had no meeting of our Society since our last mal-practice verdict, but it is a *very general* agreement among our medical men, that we will not attend any surgical cases liable to a bad result without sufficient counsel for *defense,* and the patient must pay the *cost.* The people by the juries are educating us to protect ouselves, and we propose to profit by their teachings, and let *them pay the bills.*

We shall go into malpractice suits *armed.*

Fredonia, Dunkirk, Mayville and Westfield are a *unit* upon the subject.

If we could get the Pennsylvania law to make *contracts* between *surgeons and patients binding*, we could take care of ourselves. Now a contract in this state is *annulled* by a mal-practice suit. A special statute instructing courts that such contracts were valid would rectify the whole matter. I thank you for your interest in our professional matters, which are also those of the whole state, only *we now* are in the center of the epidemic.

Yours, truly, * * * *

